# The mediating role of depressive, anxiety, and physical symptoms on work ability index in employed women with breast cancer: a prospective study from Croatia

**DOI:** 10.3325/cmj.2024.65.101

**Published:** 2024-04

**Authors:** Ivana Prga Borojević, Ines Amber Kincaid, Martina Bago, Tajana Prga Bajić, Ružica Valent, Klaudia Knezić, Bojana Knežević, Darko Marčinko

**Affiliations:** 1 University of Zagreb School of Medicine, Zagreb, Croatia; 2 Aquilonis d.o.o., Zagreb, Croatia; 3 Andrija Štampar Teaching Institute of Public Health, Zagreb, Croatia; 4 Sveti Ivan University Psychiatric Hospital, Zagreb, Croatia; 5 Department for Quality Assurance and Improvement in Healthcare, University Hospital Center Zagreb, Zagreb, Croatia; 6 Department of Surgery, Zagreb University Hospital Center, Zagreb, Croatia; 7 Department of Psychiatry and Psychological Medicine, Zagreb University Hospital Center, Zagreb, Croatia

## Abstract

**Aim:**

To explore the relationship between the current work ability index (WAI) and depressive and anxiety symptoms in breast cancer (BC) patients and the role of depressive, anxiety, and physical symptoms in mediating this relationship.

**Methods:**

This prospective study enrolled 83 employed women with BC. At baseline assessment (in the first three months following BC diagnosis) and follow-up assessment (one year after baseline), participants completed the WAI, Beck Depression Inventory-II, State-Trait Anxiety Inventory, and European Organisation for Research and Treatment of Cancer Quality of Life Questionnaire with a breast cancer-specific module. Mediation analyses were conducted to explore the mechanism by which depressive, anxiety, and physical symptoms influenced the relationship between WAI and depressive and anxiety symptoms.

**Results:**

WAI was negatively associated with depressive and anxiety symptoms. The effect of baseline depressive and trait anxiety symptoms on WAI at follow-up was mediated by both depressive and trait anxiety symptoms, as well as by physical symptoms at follow-up. The effect of baseline state anxiety symptoms on WAI at follow-up was mediated only by state anxiety symptoms at follow-up.

**Conclusions:**

Baseline depressive and anxiety symptoms affect WAI at follow-up not only through persisting depressive and anxiety symptoms observed at follow-up but also through physical symptoms at follow-up. This indicates that efforts aimed at improving psychological health may result in simultaneous improvements in both psychological and physical health, as well as the resulting WAI.

Breast cancer is the most diagnosed cancer worldwide, with 2 261 419 new cases in 2020 (1). The survival rate has significantly improved due to the impact of screening programs, improved diagnosis, and more effective treatments (2). However, it remains lower in developing regions, such as Croatia (2). Breast cancer patients are more likely to experience depression and anxiety compared with women with no prior cancer, which negatively affects disease management and health outcomes (3). Recent meta-analyses showed that the prevalence of depression and anxiety among breast cancer patients was up to 32.2% and 41.9%, respectively (4,5).

The increasing incidence of breast cancer patients among working-age women has highlighted the importance of workforce reintegration, making it a public health challenge (6). The work ability concept, introduced in 1981 (7), is defined as a balance between job demands, work environments, and an individual’s physical and mental resources (8). Work ability is a strong predictor of return to work (9) and a critical factor for successful workplace reintegration in the cancer population (10,11). Work ability in breast cancer patients is lower upon diagnosis (12,13), and although it gradually improves over time (13-15), it remains lower years later (16,17) and compared with cancer-free controls (18,19). Work ability was previously mostly assessed with a single item from the Work Ability Index (WAI) questionnaire, the Work Ability Score (WAS). However, lower work ability among women was more frequently observed when using the WAI questionnaire than the WAS (20), suggesting that using the WAI questionnaire provides a holistic view of a participant’s work ability, capturing nuances that a single item may miss.

Research on work ability among breast cancer patients has usually focused on clinical status, physical health, work environment, individual characteristics, and societal factors (21) rather than exploring depressive and anxiety symptoms (22), which have been found to be associated with work ability in the cancer population (23). Previous studies have mostly reported a negative association between depressive symptoms and work ability, whereas the association between anxiety symptoms and work ability was predominantly found to be non-significant (23). Nonetheless, there are studies that did not observe the association between depressive symptoms and work ability (8) and, conversely, studies that did observe the association between anxiety symptoms and work ability (22,24). As the issue has so far been addressed mostly through cross-sectional surveys, it is important to provide longitudinal data to enhance understanding of the relationship between the WAI and depressive and anxiety symptoms in breast cancer patients. It is also important to understand whether this relationship is moderated by other factors, such as physical symptoms, which have been associated with a variety of work outcomes, including return to work, employment, and work ability (23-25).

To the best of our knowledge, no study so far has investigated the association between the WAI and depressive and anxiety symptoms in the cancer population using the WAI questionnaire, the Beck Depression Inventory-II (BDI-II), and the State-Trait Anxiety Inventory (STAI). The aim of this study was to examine the association between the WAI and depressive and anxiety symptoms and the possible role of depressive, anxiety, and physical symptoms in mediating the relationship between the WAI and depressive and anxiety symptoms in breast cancer patients.

## PARTICIPANTS AND METHODS

### Participants

The inclusion criteria for this study were a new diagnosis of stage I-III breast cancer, age 18-60, and being employed at the time of diagnosis. We specifically targeted employed women under 60, although the retirement age in Croatia is 65, to investigate the WAI in those expected to have productive work years following cancer treatment. All participants had undergone prior therapy (surgery, neoadjuvant chemotherapy, or surgery with chemotherapy) before the baseline assessment. Non-inclusion criteria were a prior cancer diagnosis (to focus on the impact of first-time breast cancer), stage-IV breast cancer (due to extensive treatment and poorer prognosis), history of psychotic disorders (to ensure the validity of psychological assessments), and unemployment (to examine the current WAI of employed women). The exclusion criteria at follow-up were stage-IV breast cancer, retirement, palliative treatment, and the diagnosis of psychotic disorders after cancer diagnosis. Participants (n = 181) meeting the inclusion criteria were approached for baseline assessment. The sample size at baseline was 114 (response rate: 63%) and 85 at follow-up (drop-out rate: 25%). The reasons for study participants (n = 29) not to participate in follow-up were loss to follow-up (n = 25) and declining to participate after a reminder telephone call (n = 4). Two out of 85 participants at follow-up were excluded due to retirement. The final analysis was conducted on a convenience sample of 83 participants.

With the power value set at 0.80 and the indirect effect in the mediation analysis tested with the percentile bootstrap confidence interval, the sample of 78 participants was sufficient to detect significant effects if both the *a* and *b* coefficients were of medium size or larger (26).

### Methods

This prospective study was conducted at the Zagreb University Hospital Center. Data were collected in the first three months after breast cancer diagnosis (baseline) and one year after the baseline (follow-up). The interval between baseline and follow-up was one year (mean = 1.0; standard deviation = 0.07). Eligible participants were approached from May 2021 to May 2022 for the baseline assessment and were given written information about the study. Additionally, eligible participants were informed that they would be contacted for a follow-up, and the procedures for such follow-up interactions were clearly outlined. After fully informed consent was obtained from all individual participants included in the study, participants completed the following questionnaires at baseline: the WAI, BDI-II, STAI, and European Organisation for Research and Treatment of Cancer Quality of Life Questionnaire-C30 version 3 (QLQ-C30) with the breast cancer-specific module (QLQ-BR23). Demographic, socioeconomic, and clinical data were also collected. For participants who participated at baseline, the same questionnaires were sent via post 12 months later. A pre-stamped, addressed envelope was included for ease of return. Participants not returning the survey within a month received reminder telephone calls and were considered lost to follow-up after three unreturned calls. This study was conducted in line with the principles of the Declaration of Helsinki. It was approved by the Ethics Committee of the University Hospital Centre Zagreb and the School of Medicine, University of Zagreb.

### Measures

Demographic and socioeconomic data were collected using a self-reported questionnaire designed for this study. The questionnaire gathered information about age, marital status, parenthood, household status, educational level, employment status, and monthly household income. Clinical data on the stage of breast cancer and therapy were obtained from the medical records.

Depressive symptoms were assessed with the BDI-II (27), a self-reported instrument aligned with the Diagnostic and Statistical Manual of Mental Disorders, Fourth Edition (DSM-IV). The BDI consists of 21 items rated on a four-point scale, categorizing depression into minimal (0-13), mild (14-19), moderate (20-28), and severe (29-63) (27).

Anxiety symptoms were assessed with the STAI (28), a 40-item self-evaluation questionnaire that includes measures of state and trait anxiety. The State-Anxiety Scale (STAI-S) consists of twenty statements that evaluate the respondents’ current anxiety levels, while the Trait-Anxiety Scale (STAI-T) consists of twenty statements that assess general anxiety feelings. A four-point rating indicates high-level anxiety, while one indicates absence. The scale ranges from 20 to 80, with a score of 40 or higher indicating clinically meaningful anxiety (28).

Physical symptoms were assessed with a composite measure of physical symptoms (the Physical Symptoms Measure, PSM). Although participants completed the entire QLQ-C30 and the QLQ-BR23, our analysis focused solely on the derived PSM to assess symptom severity. The PSM was derived from six symptom scales (fatigue, nausea and vomiting, pain, systemic therapy side effects, breast symptoms, and arm symptoms) and five single items related to various physical symptoms (dyspnea, insomnia, appetite loss, constipation, and diarrhea) from the QLQ-C30 and QLQ-BR23 (29). The score on the PSM is an average of the scores on these eleven scales or single items, ranging from 0 to 100, with a score of 0 indicating no physical symptoms and a score of 100 indicating the presence of all the assessed symptoms (30).

The WAI was assessed with the self-evaluation WAI questionnaire (31). This instrument consists of seven items: current work ability compared with lifetime best, job demands, current diseases, estimated work impairment, sick leave, prognosis of work ability in two years, and mental resources. The score ranges from 7 to 49 points and is categorized into four levels: poor (7-27), moderate (28-36), good (37-43), and excellent (44-49) (31).

### Statistical analysis

**Figure 1:**
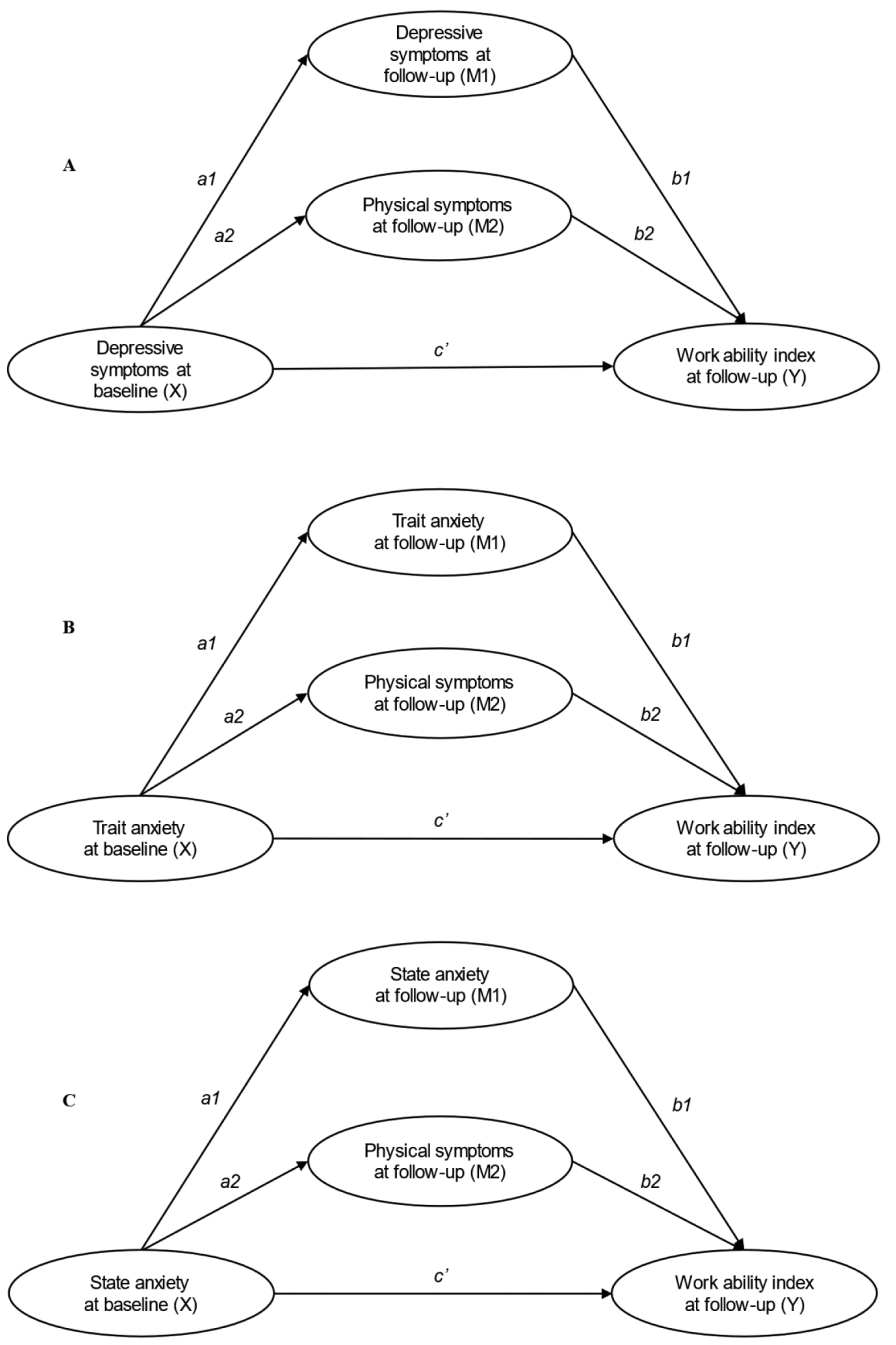
**(A)** Mediation model with work ability index (**Y**) at follow-up as the outcome, depressive symptoms (**X**) at baseline as the predictor, and depressive symptoms (M1) and physical symptoms (M2) at follow-up as the mediators. **(B)** Mediation model with work ability index (**Y**) at follow-up as the outcome, trait anxiety (**X**) at baseline as the predictor, and trait anxiety (M1) and physical symptoms (M2) at follow-up as the mediators. **(C)** Mediation model with work ability index (**Y**) at follow-up as the outcome, state anxiety (**X**) at baseline as the predictor, and state anxiety (M1) and physical symptoms (M2) at follow-up as the mediators. *Abbreviations: X – predictor; Y – outcome; M1 – mediator 1; M2 – mediator 2; a1, a2, b1, b2 – regression coefficients defining indirect effects of mediators 1 and 2; c' – direct effect of the predictor at baseline on the outcome at follow-up.

Descriptive statistics were used to summarize all variables: frequencies for categorical variables, and mean, standard deviation, median, and interquartile range for continuous variables. The normality of distribution for continuous variables was tested with the Shapiro-Wilk test, along with measures of skewness and kurtosis. The reliability of the BDI-II, STAI, and WAI questionnaires was assessed with test-retest correlations and Cronbach’s alpha. The correlation between depressive, anxiety, and physical symptoms and WAI was evaluated with Pearson correlation coefficient. The statistical significance level was set at *P* ≤ 0.05. To investigate the possible mediating role of depressive, anxiety, and physical symptoms in the relationship between depressive and anxiety symptoms at baseline and WAI at follow-up, three mediation analyses were conducted using a parallel multiple mediator model (Figure 1) (32). This approach was chosen as it allows the simultaneous assessment of two mediators. All three analyses included the following five covariates measured at baseline: age, education, marital status, household income, and therapy. All regression coefficients (β) were standardized. The significance of indirect effects was evaluated using 95% percentile bootstrap confidence intervals based on 5000 bootstrap samples. Statistical analyses were conducted with IBM SPSS version 25.0 (33). Mediation analyses were performed with the PROCESS macro version 3.5 (32).

## RESULTS

Baseline characteristics of participants are presented in Table 1. The reliability of the BDI-II and the STAI was satisfactory. Cronbach’s alpha values for the BDI-II at baseline and follow-up were 0.89 and 0.91, respectively; for the STAI-T - 0.92 and 0.93, respectively; and for both measurements of the STAI-S - 0.95 (Table 2). Cronbach’s alpha was not calculated for the WAI questionnaire due to the heterogeneity of its item scales, which deviate from the tau-equivalent model. Similarly, reliability indicators were not calculated for the PSM because it is not a measure of a single latent dimension.

**Table 1:** Baseline characteristics of participants (N = 83)*

Age (years)	48.8 ± 8.23
Marital status	
married	55 (66.3)
cohabiting	3 (3.6)
single	9 (10.8)
divorced	14 (16.9)
widowed	2 (2.4)
Household size	
alone	7 (8.4)
shared household	76 (91.6)
Children	
has children	70 (84.3)
no children	13 (15.7)
Education	
elementary school	2 (2.4)
high school	39 (47.0)
vocational college	9 (10.84)
university	33 (39.76)
Employment status	
full-time employment	78 (94.0)
part-time employment	4 (4.8)
other	1 (1.2)
Income^†^	1391.2 ± 568.92
Cancer stage^‡^	
IA	54 (65.0)
IIA	24 (29.0)
IIB	3 (3.6)
IIIA	1 (1.2)
IIIC	1 (1.2)
Cancer therapy	
radical surgery	33 (39.8)
breast-conserving surgery	27 (32.6)
neoadjuvant chemotherapy	12 (14.4)
radical or breast-conserving surgery and chemotherapy	11 (13.2)

*Data are presented as n (%) or mean ± standard deviation.

†Monthly household income in euros (€).

‡Based on the TNM Classification of Malignant Tumours.**Table 2.** Descriptive statistics, measures of distribution normality and reliability (N = 83)*

The WAI score remained moderate from baseline (32.6 ± 7.43) to follow-up (31.6 ± 8.10) (Table 2). At baseline, 23% of participants had a poor WAI score, 43% had moderate, 31% good, and 2% excellent. At the follow-up, 30% of participants had a poor WAI score, 41% had moderate, 27% good, and 2% excellent. Depressive symptoms were minimal, with mean scores of 7.9 ± 6.80 at baseline and 9.0 ± 7.39 at follow-up (Table 2). The mean scores for trait and state anxiety were 39.9 ± 9.56 and 41.5 ± 12.72 at baseline, and 41.0 ± 9.85 and 41.1 ± 11.46 at follow-up, respectively (Table 2). Clinically significant symptoms of depression, trait anxiety, and state anxiety were observed in 17%, 49%, and 51% of participants at baseline, respectively, and in 22%, 52%, and 52% at follow-up. The PSM score indicated that breast cancer patients experienced a low level of physical symptoms, with a mean score of 19.8 ± 14.04 at baseline and a median score of 17.7 (IQR 16.1) at follow-up (Table 2).

### Intercorrelations

Most variables were significantly correlated (Table 3). Baseline WAI was significantly and negatively correlated with depressive symptoms (baseline: *r* = -0.47, *P* < 0.001; follow-up: *r* = -0.41, *P* < 0.001), trait anxiety (baseline: *r* = -0.39, *P* < 0.001; follow-up: *r* = -0.33, *P* = 0.002), state anxiety (baseline: *r* = -0.25, *P* = 0.021; follow-up: *r* = -0.29, *P* = 0.008), and physical symptoms (baseline: *r* = -0.51, *P* < 0.001; follow-up: *r* = -0.51, *P* < 0.001). WAI at follow-up was significantly and negatively correlated with depressive symptoms (baseline: *r* = -0.42, *P* < 0.001; follow-up: *r* = -0.58, *P* < 0.001), trait anxiety (baseline: *r* = -0.42, *P* < 0.001; follow-up: *r* = -0.52, *P* < 0.001), and physical symptoms (baseline: *r* = -0.50, *P* < 0.001; follow-up: *r* = -0.68, *P* < 0.001), as well as with state anxiety at follow-up (*r* = -0.51, *P* < 0.001), but not at baseline (*r* = -0.20, *P* = 0.069).

**Table 3:** Intercorrelations between work ability index, depressive symptoms, trait anxiety, state anxiety, physical symptoms, income at baseline and at follow-up, and age at baseline (N = 83)*

	(1)	(2)	(3)	(4)	(5)	(6)	(7)	(8)	(9)	(10)	(11)	(12)
(1) Work ability index^†^												
(2) Work ability index^‡^	0.60*											
(3) Age^†^	-0.17	-0.30*										
(4) Depressive symptoms^†^	-0.47*	-0.42*	0.17									
(5) Depressive symptoms^‡^	-0.41*	-0.58*	0.08	0.63*								
(6) Trait anxiety^†^	-0.39*	-0.42*	0.30*	0.69*	0.54*							
(7) Trait anxiety^‡^	-0.33*	-0.52*	0.25*	0.56*	0.77*	0.73*						
(8) State anxiety^†^	-0.25*	-0.20	0.26*	0.67*	0.33*	0.68*	0.46*					
(9) State anxiety^‡^	-0.29*	-0.51*	0.17	0.52*	0.74*	0.68*	0.83*	0.52*				
(10) Physical symptoms^†^	-0.51*	-0.50*	0.16	0.69*	0.46*	0.46*	0.35*	0.43*	0.32*			
(11) Physical symptoms^‡^	-0.51*	-0.68*	0.08	0.57*	0.70*	0.41*	0.49*	0.25*	0.52*	0.60*		
(12) Income^†^	0.12	0.28*	-0.25*	-0.04	-0.05	-0.16	-0.08	-0.20	-0.12	-0.12	-0.01	
(13) Income^‡^	0.12	0.31*	-0.17	-0.06	-0.10	-0.09	-0.03	-0.14	-0.14	-0.12	-0.07	0.85*

*Intercorrelations are significant at *P* ≤ 0.05.

†Variable measured at baseline.

‡Variable measured at follow-up.

### Relationship between work ability index and depressive, anxiety, and physical symptoms

The study revealed a significant indirect effect of baseline depressive symptoms on WAI at follow-up through depressive symptoms (a1b1; β = -0.15, 95% CI -0.31 to -0.02) and physical symptoms (a2b2; β = -0.28, 95% CI -0.50 to -0.08) at follow-up. The direct effect of baseline depressive symptoms on WAI at follow-up was not significant (c’; β = 0.03, *P* = 0.784) (Table 4). Similarly, baseline trait anxiety had a significant indirect effect on WAI at follow-up through trait anxiety (a1b1; β = -0.16, 95% CI -0.38 to -0.03) and physical symptoms (a2b2; β = -0.23, 95% CI -0.39 to -0.07) at follow-up. The direct effect of baseline trait anxiety on WAI at follow-up was not significant (c’; β = 0.07, *P* = 0.557) (Table 4). For baseline state anxiety, a significant indirect effect on WAI at follow-up was observed through follow-up state anxiety (a1b1; β = -0.13, 95% CI -0.26 to -0.04), while the indirect effect through physical symptoms at follow-up was not observed (a2b2; β = -0.12, 95% CI -0.29 to 0.02). The direct effect of baseline state anxiety on WAI at follow-up was not significant (c’; β = 0.14, *P* = 0.115) (Table 4).

**Table 4:** Standardized regression coefficients for the specific indirect effect through mediator 1, specific indirect effect through mediator 2, and the direct effect of the predictor on the outcome work ability index at follow-up for three mediation analyses (N = 83)*

Mediation analysis	Predictor	Regression coefficient	β	p	95% CI^†^	
					BootLL	BootUL
Depressive symptoms	Depressive symptoms^‡^	*a1b1* depressive symptoms^§^	-0.15		-0.31	-0.02
		a2b2 physical symptoms^§^	-0.28		-0.50	-0.08
		c'	0.03	0.784		
		a1	0.66	<0.001		
		b1	-0.22	0.058		
		a2	0.56	<0.001		
		b2	-0.50	<0.001		
Trait anxiety	Trait anxiety^‡^	a1b1 trait anxiety^§^	-0.16		-0.38	-0.03
		a2b2 physical symptoms^§^	-0.23		-0.39	-0.07
		c'	0.07	0.557		
		a1	0.72	<0.001		
		b1	-0.22	0.064		
		a2	0.40	<0.001		
		b2	-0.57	<0.001		
State anxiety	State anxiety^‡^	a1b1 state anxiety^§^	-0.13		-0.26	-0.04
		a2b2 physical symptoms^§^	-0.12		-0.29	0.02
		c'	0.14	0.115		
		a1	0.52	<0.001		
		b1	-0.25	0.013		
		a2	0.22	0.059		
		b2	-0.55	<0.001		

*Abbreviations: β – standardized regression coefficient; CI – confidence interval; BootLL – lower limit; BootUL – upper limit; a1b1 – specific indirect effect through mediator 1; a2b2 – specific indirect effect through mediator 2; c' – direct effect of the predictor on the outcome; a1, a2, b1, b2 – regression coefficients defining indirect effects of mediators 1 and 2.

†The indirect effect was significant at *P* < 0.05 if zero was not included in the 95% confidence interval for that indirect effect.

‡Variable measured at baseline.

§Variable measured at follow-up.

## DISCUSSION

Employing a combination of assessment tools, including the WAI questionnaire, BDI-II, and STAI, this prospective study explored the relationship between the WAI and depressive and anxiety symptoms in breast cancer patients in Croatia. This contribution to the literature provides a deeper understanding of the observed relationship, given that research on work ability in breast cancer patients in Eastern Europe is limited compared with findings from Western countries. By focusing on a Croatian cohort, this study addresses this research gap.

Within the first year after diagnosis, patients in this study had a moderate WAI score, which aligns with previous research (8,13-15) and a low level of physical symptoms, which may be explained by the significant presence (94%) of early-stage cancer (IA and IIA) in our sample (Table 1). Additionally, the observed mean scores for depressive and anxiety symptoms closely matched the pooled mean scores for anxiety and depression among breast cancer patients measured by the STAI and BDI in recent meta-analyses (4,5).

Our research corroborates prior studies that found a negative association between WAI and depressive symptoms (23,34,35). However, it diverges from previous findings (23) as we observed a negative association between WAI and anxiety symptoms. Additionally, our research revealed a positive association between physical symptoms and depressive and anxiety symptoms, which aligns with the literature that highlights the prevalence of this symptom cluster and the necessity for an integrated care approach (36,37). We also found that higher levels of physical symptoms were associated with a lower WAI, which supports the conclusions from earlier studies (12,24,34,35). Cancer patients with a lower symptom burden and higher work ability are more likely to continue working after diagnosis and treatment (38).

Mediation analysis revealed that higher baseline depressive or trait anxiety symptoms were associated with lower WAI at follow-up, mediated by both depressive or trait anxiety and physical symptoms at follow-up. Existing literature strongly suggests that emotional functioning is associated with physical health (40,41). An important question is whether physical symptoms are related to emotions such as anxiety or depression or to emotion regulation. As a construct, emotion includes feeling, motor expression, and physiological activation. The physiological response to an emotion depends on the regulation strategy employed, whether adaptive or maladaptive (41,42). Adaptive emotion regulation strategies are positively associated with physical health (43,44) and with a decrease in depression and anxiety (45,46). Conversely, maladaptive emotion regulation strategies are associated with a decline in physical health (43,44) and a long-term increase in anxiety and depression (45,46). A recent review has emphasized the importance of emotion regulation in breast cancer management (47). Breast cancer patients using less adaptive emotion regulation strategies have more depressive and anxiety symptoms (48). Furthermore, emotion dysregulation may exacerbate physical symptoms in breast cancer patients (49). Therefore, it is possible that emotions may “contribute” to an increase in physical symptoms only when a maladaptive emotion regulation strategy is used. Our findings indicated that only state anxiety at follow-up mediated the relationship between baseline state anxiety and WAI at follow-up, in contrast to the mediation analyses involving trait anxiety and depressive symptoms, where physical symptoms at follow-up also acted as mediators. This distinction may be attributed to the nature of state anxiety’s impact on physical functioning being potentially less intense and more transient compared with that of trait anxiety symptoms, despite the general expectation that all emotions affect physical functioning (41).

The study has several limitations, including the potential sampling bias due to the convenience sample used, which may lead to an incorrect population mean estimation. The response rate was 63%, with a 25% drop-out rate after one year, resulting in selection bias. The study focused on employed breast cancer patients, which raises questions about its generalizability to non-working patients. Additionally, the study did not assess depressive, anxiety, or physical symptoms pre-diagnosis, and no measures of emotion regulation were applied. Finally, we considered only two time points; however, it could be useful to monitor the association between WAI and depressive and anxiety symptoms over time to provide a more nuanced understanding of the relationship observed.

In conclusion, our findings indicate that baseline depressive and anxiety symptoms impact WAI at follow-up, not only through depressive and anxiety symptoms but also through physical symptoms at follow-up. This strengthens our understanding of the complex relationship between depressive, anxiety, and physical symptoms and WAI, all of which have been identified as factors in work-related studies in breast cancer populations. Research emphasizes the importance of routine mental health screening after diagnosis, as impaired WAI may be concealed by unrecognized baseline depressive and anxiety symptoms that worsen over time. Furthermore, tailoring targeted interventions and timely identification of treatment-related symptoms can provide comprehensive care that considers both mental and physical aspects, significantly impacting the WAI of breast cancer patients.

**Table:** 

	M	sd	Md	IQR	min	max	α	test-retest r	skewness	kurtosis	SW (df = 83)	SW p
BDI-II^†^	7.9	6.80	6	9	0	30	0.888	0.63^§^	1.25	1.19	0.88	<0.001
BDI-II^‡^	9.0	7.39	7	8	0	37	0.907		1.28	1.64	0.89	<0.001
STAI-T^†^	39.9	9.56	39	11	23	70	0.920	0.73^§^	0.53	0.29	0.97	0.056
STAI-T^‡^	41.0	9.85	41	10	21	68	0.930		0.48	0.41	0.98	0.107
STAI-S^†^	41.5	12.72	40	15	21	74	0.949	0.52^§^	0.61	-0.06	0.96	0.009
STAI-S^‡^	41.1	11.46	40	15	20	70	0.948		0.33	-0.36	0.98	0.291
WAI^†^	32.6	7.43	33	9	10	46		0.60^§^	-0.80	0.47	0.96	0.006
WAI^‡^	31.6	8.10	33	12.5	10	46			-0.56	-0.30	0.96	0.017
PSM^†^	19.8	14.04	17	20.7	0	59			0.92	0.45	0.93	<0.001
PSM^‡^	20.0	13.35	17.7	16.1	0	68			1.20	2.06	0.92	<0.001
Age^†^	48.8	8.23	50.2	10.4	21.4	60.9			-0.92	0.72	0.94	<0.001
Income^†^	1391.2	568.92	1460.0	796.3	0	2256.0			-0.03	-0.85	0.95	0.002

*Abbreviations: M – mean; sd – standard deviation; Md – median; IQR – interquartile range; min – minimum sample result; max – maximum sample result; α – Cronbach’s alpha; test-retest r – Pearson correlation between baseline and follow-up; SW – Shapiro Wilk test statistic value; SW p – Shapiro-Wilk test significance level; BDI-II – Beck Depression Inventory-II; STAI-T – trait subscale of the State-Trait Anxiety Inventory; STAI-S – state subscale of the State-Trait Anxiety Inventory; WAI – Work Ability Index questionnaire; PSM – Physical Symptoms Measure.

†Variable measured at baseline.

‡Variable measured at follow-up.

§Results of test-retest *r* are significant at *P* < 0.01.

## References

[1] Sung H Ferlay J Siegel RL Laversanne M Soerjomataram I Jemal A Global cancer statistics 2020: GLOBOCAN estimates of incidence and mortality worldwide for 36 cancers in 185 countries. CA Cancer J Clin 2021 71 209 49 10.3322/caac.21660 33538338

[2] Dafni U Tsourti Z Alatsathianos I Breast cancer statistics in the European Union: incidence and survival across European countries. Breast Care (Basel) 2019 14 344 53 10.1159/000503219 31933579 PMC6940474

[3] Carreira H Williams R Müller M Harewood R Stanway S Bhaskaran K Associations between breast cancer survivorship and adverse mental health outcomes: a systematic review. J Natl Cancer Inst 2018 110 1311 32 10.1093/jnci/djy177 30403799 PMC6292797

[4] Pilevarzadeh M Amirshahi M Afsargharehbagh R Rafiemanesh H Hashemi SM Balouchi A Global prevalence of depression among breast cancer patients: a systematic review and meta-analysis. Breast Cancer Res Treat 2019 176 519 33 10.1007/s10549-019-05271-3 31087199

[5] Hashemi SM Rafiemanesh H Aghamohammadi T Badakhsh M Amirshahi M Sari M Prevalence of anxiety among breast cancer patients: a systematic review and meta-analysis. Breast Cancer 2020 27 166 78 10.1007/s12282-019-01031-9 31828585

[6] Dumas A Luis IV Bovagnet T El Mouhebb M Di Meglio A Pinto S Impact of Breast Cancer Treatment on Employment: Results of a Multicenter Prospective Cohort Study (CANTO). J Clin Oncol 2020 38 734 43 10.1200/JCO.19.01726 31834818 PMC7048162

[7] Ilmarinen J Work ability—a comprehensive concept for occupational health research and prevention. Scand J Work Environ Health 2009 35 1 5 10.5271/sjweh.1304 19277432

[8] Silvaggi F Mariniello A Leonardi M Silvani A Lamperti E Di Cosimo S Psychosocial factors associated with workability after surgery in cancer survivors: An explorative study. J Health Psychol 2023 28 999 1010 10.1177/13591053231151286 36800903 PMC10492438

[9] Wolvers MDJ Leensen MCJ Groeneveld IF Frings-Dresen MHW De Boer AGEM Predictors for earlier return to work of cancer patients. J Cancer Surviv 2018 12 169 77 10.1007/s11764-017-0655-7 29076003 PMC5884890

[10] Boelhouwer IG Vermeer W van Vuuren T Late effects of cancer (treatment) and work ability: guidance by managers and professionals. BMC Public Health 2021 21 1 7 10.1186/s12889-021-11261-2 34187437 PMC8240423

[11] Hou W Li Q Liu X Zeng Y Cheng AS Exploring the employment readiness and return to work status of breast cancer patients and related factors. Int J Nurs Sci 2021 8 426 31 10.1016/j.ijnss.2021.09.001 34631993 PMC8488802

[12] Magnavita N Di Prinzio RR Meraglia I Vacca ME Arnesano G Merella M Supporting return to work after breast cancer: a mixed method study. Healthcare (Basel) 2023 11 2343 10.3390/healthcare11162343 37628540 PMC10454012

[13] Tamminga SJ Jansen LP Frings-Dresen MH de Boer AG Long-term employment status and quality of life after cancer: a longitudinal prospective cohort study from diagnosis up to and including 5 years post diagnosis. Work 2020 66 901 7 10.3233/WOR-203234 32925145 PMC7683081

[14] Gregorowitsch ML van den Bongard HJ Couwenberg AM Young-Afat DA Haaring C Van Dalen T Self-reported work ability in breast cancer survivors; a prospective cohort study in the Netherlands. Breast 2019 48 45 53 10.1016/j.breast.2019.08.004 31493582

[15] Tevaarwerk AJ Kwekkeboom K Buhr KA Dennee A Conkright W Onitilo AA Results from a prospective longitudinal survey of employment and work outcomes in newly diagnosed cancer patients during and after curative-intent chemotherapy: A Wisconsin Oncology Network study. Cancer 2021 127 801 8 10.1002/cncr.33311 33231882 PMC7945680

[16] Vandraas K Falk RS Bøhn SK Kiserud C Lie HC Smedsland SK Work ability 8 years after breast cancer: exploring the role of social support in a nation-wide survey. Breast Cancer Res Treat 2022 193 685 94 10.1007/s10549-022-06599-z 35445949 PMC9114073

[17] Boelhouwer IG Vermeer W van Vuuren T Work ability among employees 2-10 years beyond breast cancer diagnosis: Late treatment effects and job resources – A longitudinal study. Work 2023 74 1061 76 10.3233/WOR-211288 35527613

[18] Vella F Filetti V Cirrincione L Rapisarda V Matera S Skerjanc A Work ability after breast cancer: Study of healthcare personnel operating in a hospital of south Italy. Int J Environ Res Public Health 2022 19 10835 10.3390/ijerph191710835 36078550 PMC9518308

[19] Gómez-Molinero R Guil R Boosting return to work after breast cancer: The mediator role of perceived emotional intelligence. Psychooncology 2020 29 1936 42 10.1002/pon.5527 32840943

[20] El Fassi M Bocquet V Majery N Lair ML Couffignal S Mairiaux P Work ability assessment in a worker population: comparison and determinants of Work Ability Index and Work Ability score. BMC Public Health 2013 13 1 10 10.1186/1471-2458-13-305 23565883 PMC3637198

[21] Sun Y Shigaki CL Armer JM Return to work among breast cancer survivors: a literature review. Support Care Cancer 2017 25 709 18 10.1007/s00520-016-3446-1 27873016

[22] Kim SY Kissane DW Richardson G Senior J Morgan J Gregory P The role of depression and other psychological factors in work ability among breast cancer survivors in Australia. Psychooncology 2022 31 167 75 10.1002/pon.5802 34460129

[23] Tan C Yip SYC Chan RJ Chew L Chan A Investigating how cancer-related symptoms influence work outcomes among cancer survivors: a systematic review. J Cancer Surviv 2022 16 1065 78 10.1007/s11764-021-01097-5 34424498 PMC9489549

[24] Parambil NA Kannan S Work ability, anxiety, and depression among long-term breast cancer survivors of northern Kerala, India; A historical cohort study. Asian Pac J Cancer Prev 2024 25 115 22 10.31557/APJCP.2024.25.1.115 38285775 PMC10911738

[25] Fardell JE Tan SY Kerin-Ayres K Dhillon HM Vardy JL Symptom clusters in survivorship and their impact on ability to work among cancer survivors. Cancers (Basel) 2023 15 5119 10.3390/cancers15215119 37958295 PMC10647426

[26] Fritz MS MacKinnon DP Required sample size to detect the mediated effect. Psychol Sci 2007 18 233 9 10.1111/j.1467-9280.2007.01882.x 17444920 PMC2843527

[27] Beck AT, Steer RA, Brown G. Priručnik za Beckov invertar depresije-II [in Croatian]*.* Jastrebarsko: Naklada Slap; 2009.

[28] Spielberger CD, Gorsuch RL, Lushene R, Vagg PR, Jacobs GA. Priručnik za Upitnik anksioznosti kao stanja i osobine ličnosti [in Croatian]. Jastrebarsko: Naklada Slap; 2000.

[29] Aaronson NK Ahmedzai S Bergman B Bullinger M Cull A Duez NJ The European Organization for Research and Treatment of Cancer QLQ-C30: a quality-of-life instrument for use in international clinical trials in oncology. J Natl Cancer Inst 1993 85 365 76 10.1093/jnci/85.5.365 8433390

[30] Fayers P, Aaronson NK, Bjordal K, Groenvold M, Curran D, Bottomley A. The EORTC QLQ–C30 scoring manual (3rd edition). Brussels: European Organisation for Research and Treatment of Cancer; 1995.

[31] Tuomi K, Ilmarinen J, Jahkola A, Katajarinne L, Tulkki A. Work ability index. 2 ed. Helsinki: Finnish Institute of Occupational Health; 1998.

[32] Hayes AF. Introduction to mediation, moderation, and conditional process analysis: a regression-based approach. New York City: Guilford Publications; 2022.

[33] Corp IBM. IBM SPSS Statistics for Windows, Version 25.0. Armonk, NY: IBM Corp; 2017.

[34] Ho PJ Hartman M Gernaat SA Cook AR Lee SC Hupkens L Associations between workability and patient-reported physical, psychological and social outcomes in breast cancer survivors: a cross-sectional study. Support Care Cancer 2018 26 2815 24 10.1007/s00520-018-4132-2 29511953

[35] Dahl AA Fosså SD Lie HC Loge JH Reinertsen KV Ruud E Employment status and work ability in long-term young adult cancer survivors. J Adolesc Young Adult Oncol 2019 3 304 11 10.1089/jayao.2018.0109 30900929

[36] Ruiz-Casado A Alvarez-Bustos A de Pedro CG Mendez-Otero M Romero-Elia M Cancer-related fatigue in breast cancer survivors: a review. Clin Breast Cancer 2021 21 10 25 10.1016/j.clbc.2020.07.011 32819836

[37] Moloney NA Pocovi NC Dylke ES Graham PL De Groef A Psychological factors are associated with pain at all time frames after breast cancer surgery: a systematic review with meta-analyses. Pain Med 2021 22 915 47 10.1093/pm/pnaa363 33547465

[38] Duijts SF Kieffer JM van Muijen P van der Beek AJ Sustained employability and health-related quality of life in cancer survivors up to four years after diagnosis. Acta Oncol 2017 56 174 82 10.1080/0284186X.2016.1266083 28093023

[39] Krittanawong C Maitra NS Virk HUH Fogg S Wang Z Kaplin S Association of optimism with cardiovascular events and all-cause mortality: systematic review and meta-analysis. Am J Med 2022 135 856 63 10.1016/j.amjmed.2021.12.023 35123934

[40] Wei J Lu Y Li K Goodman M Xu H The associations of late-life depression with all-cause and cardiovascular mortality: The NHANES 2005–2014. J Affect Disord 2022 300 189 94 10.1016/j.jad.2021.12.104 34971700

[41] Gross JJ Emotion regulation: Current status and future prospects. Psychol Inq 2015 26 1 26 10.1080/1047840X.2014.940781

[42] Spangler DP Bell MA Deater-Deckard K Emotion suppression moderates the quadratic association between RSA and executive function. Psychophysiology 2015 52 1175 85 10.1111/psyp.12451 26018941 PMC4918628

[43] Moriarity DP Grehl MM Walsh RF Roos LG Slavich GM Alloy LB A systematic review of associations between emotion regulation characteristics and inflammation. Neurosci Biobehav Rev 2023 ••• 105162 10.1016/j.neubiorev.2023.105162 37028579 PMC10425218

[44] Cheng MY Zhang RX Wang MJ Chang MY Relationship between cognitive emotion regulation strategies and coronary heart disease: an empirical examination of heart rate variability and coronary stenosis. Psychol Health 2022 37 230 45 10.1080/08870446.2020.1859112 33435727

[45] Kraft L Ebner C Leo K Lindenberg K Emotion regulation strategies and symptoms of depression, anxiety, aggression, and addiction in children and adolescents: A meta-analysis and systematic review. Clin Psychol Sci Pract 2023 30 485 502 10.1037/cps0000156

[46] Cavicchioli M Tobia V Ogliari A Emotion regulation strategies as risk factors for developmental psychopathology: a meta-analytic review of longitudinal studies based on cross-lagged correlations and panel models. Res Child Adolesc Psychopathol. 2023 51 295 315 10.1007/s10802-022-00980-8 36208360

[47] Durosini I Triberti S Savioni L Sebri V Pravettoni G The role of emotion-related abilities in the quality of life of breast cancer survivors: a systematic review. Int J Environ Res Public Health 2022 19 12704 10.3390/ijerph191912704 36232004 PMC9566755

[48] Guimond AJ Ivers H Savard J Is emotion regulation associated with cancer-related psychological symptoms? Psychol Health 2019 34 44 63 10.1080/08870446.2018.1514462 30516396

[49] Kim JH Brigh EE Williamson TJ Krull JL Weihs K Stanton AL Transitions in coping profiles after breast cancer diagnosis: implications for depressive and physical symptoms. J Behav Med 2021 44 1 17 10.1007/s10865-020-00159-w 32535673 PMC7736058

